# Melatonin against Myocardial Ischemia-Reperfusion Injury: A Meta-analysis and Mechanism Insight from Animal Studies

**DOI:** 10.1155/2020/1241065

**Published:** 2020-06-26

**Authors:** Zhi-Jie Mao, Hui Lin, Fang-Yi Xiao, Zhou-Qing Huang, Yi-He Chen

**Affiliations:** ^1^Department of Cardiology, The First Affiliated Hospital of Wenzhou Medical University, 325000, Nanbaixiang, Wenzhou, Zhejiang, China; ^2^Department of Respiratory, The Second Affiliated Hospital and Yuying Children's Hospital of Wenzhou Medical University, 325000, Nanbaixiang, Wenzhou, Zhejiang, China

## Abstract

**Aims:**

Myocardial reperfusion damage after severe ischemia was an important issue during a clinical practice. However, the exacted pathogenesis involved remained unclear and also lacks effective interventions. Melatonin was identified to exert protective effects for alleviating the myocardial I/R injury. This meta-analysis was determined to evaluate the efficacy of melatonin treatment against reperfusion insult and further summarize potential molecular and cellular mechanisms.

**Methods and Results:**

15 eligible studies with 211 animals (108 received melatonin and 103 received vehicle) were included after searching the databases of PubMed, MEDLINE, Embase, and Cochrane. Pretreatment with melatonin was associated with a significant lower infarct size in comparison with vehicle in myocardial I/R damage (WMD: -20.45, 95% CI: -25.43 to -15.47, *p* < 0.001; *I*^2^ = 91.4%, *p* < 0.001). Evidence from subgroup analyses and sensitivity analysis indicated the robust and consistent cardioprotective effect of melatonin, while the metaregression also did not unmask any significant interactions between the pooled estimates and covariates (i.e., sample size, state, species, study type, route of administration, and duration of reperfusion, along with timing regimen of pretreatment). Accordingly, melatonin evidently increased EF (WMD: 17.19, 95% CI: 11.08 to 23.29, *p* < 0.001; *I*^2^ = 77.0%, *p* < 0.001) and FS (WMD: 14.18, 95% CI: 11.22 to 17.15, *p* < 0.001; *I*^2^ = 3.5%, *p* = 0.387) in the setting of reperfusion damage.

**Conclusions:**

Melatonin preadministration conferred a profound cardioprotection against myocardial I/R injury in preclinical studies.

## 1. Introduction

Myocardial revascularization by timely percutaneous coronary intervention (PCI) or thrombolytic therapy was the most effective therapeutic approach for saving the endangered myocardium in acute myocardial infarction. Subsequent ischemia/reperfusion (I/R) damage inevitably caused substantial loss of myocytes and thus aggravated cardiac dysfunction [1, 2]. To date, myocardial I/R injury had increasingly become a crucial issue in a clinical practice, which attracted extensive attentions of researchers. However, there was still no adjunctive pharmacologic intervention targeting myocardial reperfusion injury due to the intricate molecular and cellular mechanisms [3]. In current, emerging evidence suggested the involvement of apoptosis, platelet activation, autophagy dysfunction, or inflammatory response [4], especially excessive free radical generation in the pathophysiological process of myocardial I/R injury [5].

Accumulating evidence had demonstrated that melatonin, an important circadian hormone produced in the pineal gland, exerts cardioprotective function against a wide variety of pathologic stimuli [6]. It had also reported that patients who suffered from acute myocardial infarction and sudden cardiac death were associated with a profound lower melatonin level [7]. Notably, as a key factor in regulating the circadian rhythm, it may possibly account for the increased frequency of cardiovascular events in the early morning [8]. Numerous studies had verified the role of melatonin in alleviating the ischemia-reperfusion injury and explored the potential underlying mechanisms, which mainly focused on its powerful capacity of free radical scavenging [9–11]. Meanwhile, other researchers also found that melatonin treatment abrogated ischemia-reperfusion-induced myocardial damage by hindering the migration of neutrophil, increasing the expression of antioxidant enzymes, along with antiadrenergic actions [12–14]. This evidence further emphasized the importance of melatonin implicated in the pathogenesis of cardiac attack post myocardial I/R injury. Nonetheless, the precise biological mechanisms by which melatonin exerted its protective properties were far from being clear and thus resulted in a huge gap between experimental studies and clinical application.

Therefore, our present meta-analysis is aimed at assessing the favorable effect of melatonin in alleviating myocardial I/R injury and summarizing potential molecular and cellular mechanisms from current available evidence of animal studies.

## 2. Methods

### 2.1. Search Strategy

We conducted a literature search in PubMed, MEDLINE, Embase, and Cochrane Database from the inception to December 2018 for potentially relevant articles reporting the cardioprotection of melatonin in myocardial I/R injury. The search terms are “myocardial ischemia/reperfusion injury” OR “myocardial I/R injury” OR “myocardial ischemia-reperfusion injury” AND “melatonin”. The language was not restricted to English. Manual review of meeting abstracts, comments, and review articles was undertaken for additional citations.

### 2.2. Inclusion and Exclusion Criteria

Studies were eligible for inclusion if they met the following criteria: (a) reporting infarct size, expressed as the percentage of infarct area over area at risk (AAR), (b) melatonin compared with vehicle treatment, (c) *in vivo* or *ex vivo* animal studies, and (d) without cardiovascular-related comorbidity (i.e., obesity, diabetes, or chronic intermittent condition). We excluded studies only investigating the melatonin-mediated cardioprotection *in vitro.*

### 2.3. Data Extraction

Two investigators (Zhi-Jie Mao and Hui Lin) independently extracted the data sets related to baseline information of included studies (i.e., author, year, state, and sample size), characteristic feature of animals (i.e., species, strain, body weight, or age), and detailed therapeutic strategy (i.e., dosage, route, and timing of melatonin treatment), along with the methods for determining the infarct size of the heart post myocardial I/R injury.

### 2.4. Quality Assessment

The quality of included studies was assessed and scored by two investigators (Hui Lin and Fang-Yi Xiao) based on published criteria [15]. Peer-reviewed publication, random allocation to groups, blinded assessment of outcome, sample size calculation, compliance with animal welfare regulations, and a statement of a potential conflict of interest were scored as one point, respectively. Discrepancies were resolved by discussion with a third investigator (Zhou-Qing Huang).

### 2.5. Statistical Analysis

Continuous variables as the mean and standard deviation were used for this meta-analysis. The weighted mean difference (WMD) with the related 95% CIs was calculated using the DerSimonian and Laird random-effects approach for infarct size. Heterogeneity was evaluated by *Q* statistics and quantified using *I*^2^ statistics. Publication bias was visually assessed with a funnel plot and further detected by Begger's and Egger's tests. If significant heterogeneity (*p* < 0.10) was found across the studies, sensitivity analysis achieved by removing each study in turn, *post hoc* subgroup analyses (i.e., species, study type, duration of reperfusion, and timing regimen of pretreatment), and univariable metaregressions (i.e., sample size, state, species, study type, route of administration, and duration of reperfusion, along with timing regimen of pretreatment) were proposed to explore the potential sources of heterogeneity. *p* < 0.05 were considered statistically significant for all results but heterogeneity. Statistical analyses and graphs were done with STATA version 12.0 (STATA Corporation, College Station, TX, USA).

## 3. Results

**Figure 1:**
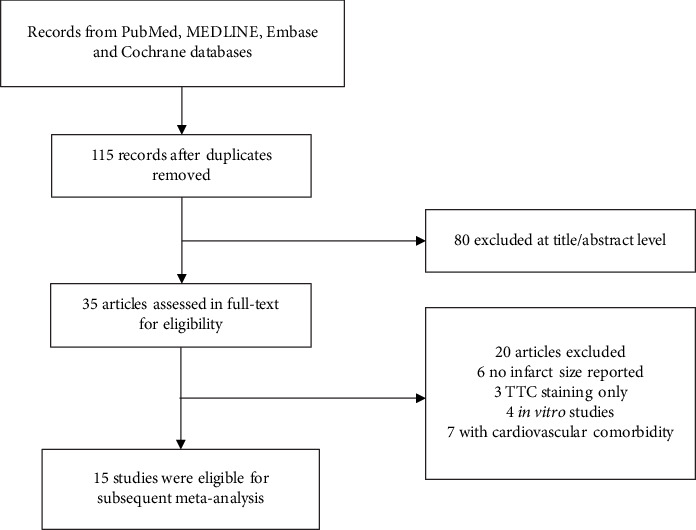
Flow diagram of the study inclusion.

Our search identified 115 study reports, of which 80 were excluded after title and abstract screening. 20 articles were relevant for full-text review (online Table 1), and 15 studies of 211 animals (108 in the melatonin treatment group and 103 in the control group) fulfilled the prespecified inclusion criteria and were finally retained for our meta-analysis (Figure 1). A comprehensive list of individual studies is shown in Table 1. 11 studies established a conventional *in vivo* myocardial I/R injury model, whereas in 4 studies, *ex vivo* regional ischemia-reperfusion was induced by left coronary artery occlusion in the perfused heart with Langendorff mode. All the studies investigated the cardioprotective effect of melatonin by using rats (either Sprague-Dawley or Wistar) and mice (C57BL/6). Infarct size was determined with Evans blue/TTC double staining in most studies; fluorescent particles, methylene blue, or blue dye added with TTC was the substitute in the remaining 4 studies. Melatonin was administered orally or by intravenous or intraperitoneal injection, along with perfusion before myocardial I/R injury. Studies were reported between 2000 and 2018. Nine of 15 studies were conducted in China, 3 studies in South Africa, 2 studies in Turkey, and 1 study in France. The quality of included studies is assessed in Table 2, with the majority of studies scoring from 2 to 4, indicating reliable data and low risk of bias. The molecular mechanism underlying the beneficial effect of melatonin in protecting the heart against myocardial I/R insult was sophisticated and diverse; it is summarized in Table 3.

### 3.1. Infarct Size

**Figure 2:**
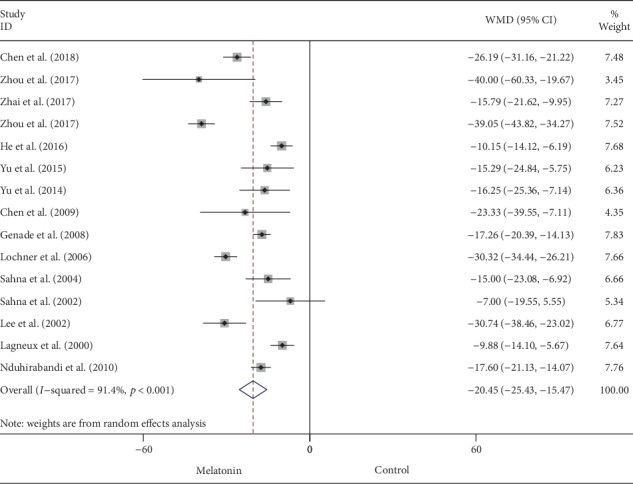
Summary WMD of infarct size for melatonin pretreatment versus vehicle in myocardial I/R injury.

**Figure 3:**
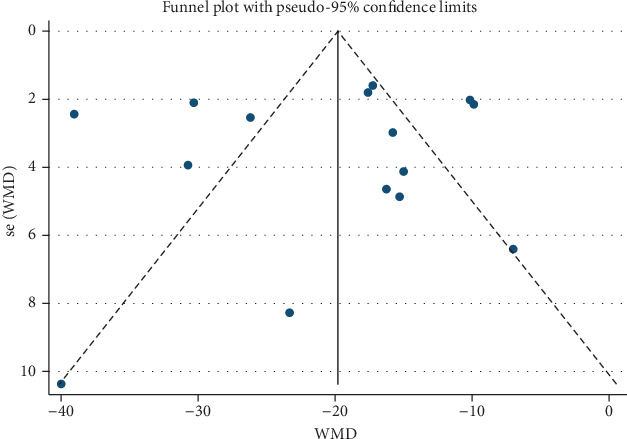
Funnel plot for assessment of publication bias among the included studies.

As presented in Figure 2, pretreatment with melatonin significantly reduced the infarct size in comparison with vehicle treatment (WMD: -20.45, 95% CI: -25.43 to -15.47, *p* < 0.001). There was a significant amount of heterogeneity across the studies (*I*^2^ = 91.4%, *p* < 0.001) (Figure 3). A symmetrical funnel plot followed with Begg's (*p* = 0.79) and Egger's (*p* = 0.711) tests reasonably excluded the presence of publication bias. Moreover, sensitivity analysis did not reveal any variation in the pooled estimate of WMD, supporting the robust effect in favor of melatonin treatment (Table 4). Post hoc subgroup analyses performed to explore the source of heterogeneity among studies did not show significant results (Table 5). Univariable metaregression failed to expose any significant correlation between study-level covariates, i.e., sample size, species, study type, state, route of administration, reperfusion duration, and timing regimen of pretreatment and the magnitude of WMD (Table 6).

### 3.2. Cardiac Function

**Figure 4:**
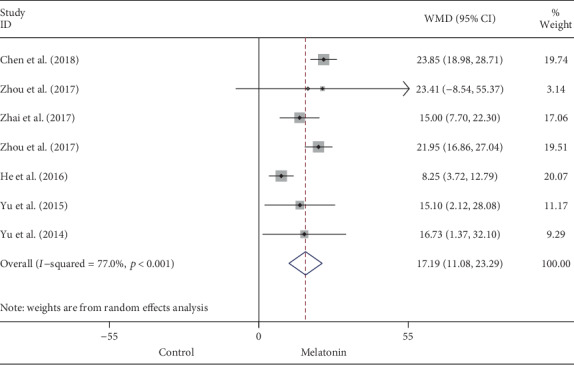
Summary WMD of EF for melatonin pretreatment versus vehicle in myocardial I/R injury.

**Figure 5:**
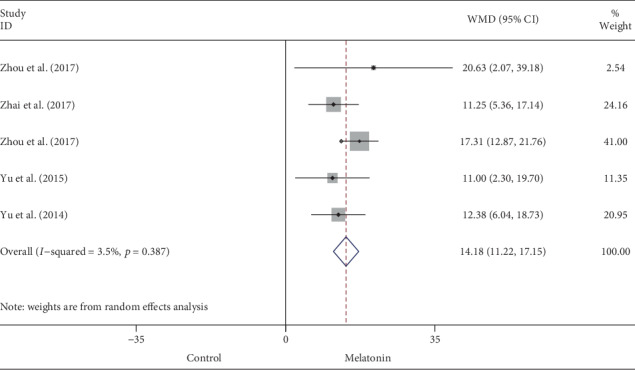
Summary WMD of FS for melatonin pretreatment versus vehicle in myocardial I/R injury.

Data on left ventricular EF was available by echocardiography in 7 out of 15 studies. Melatonin treatment was associated with significantly higher EF after myocardial I/R injury (WMD: 17.19, 95% CI: 11.08 to 23.29, *p* < 0.001) (Figure 4), with high heterogeneity (I^2^ = 77.0%, *p* < 0.001). FS was evaluated in 5 eligible studies; in accordant with EF, melatonin administration evidently increased FS (WMD: 14.18, 95% CI: 11.22 to 17.15, *p* < 0.001) (Figure 5), with no significant heterogeneity (*I*^2^ = 3.5%, *p* = 0.387). Systematically removing each study also did not markedly affect the pooled WMD and related *p* value, respectively.

## 4. Discussion

As far as we know, it was the first study to pool all available evidence and show the beneficial effect of melatonin in protecting the myocardium against ischemia/reperfusion damage. A previous meta-analysis confirmed the markedly neuroprotective effect of melatonin in improving the outcomes in the animal models of focal cerebral ischemia [16], while our meta-analysis demonstrated that melatonin treatment was associated with a significantly reduced infarct size in the context of myocardial I/R injury. Accordingly, similar improvement was also noted in left ventricular EF and FS, which indicated the critical role of melatonin in attenuating the reperfusion injury and subsequent cardiac dysfunction.

Although acute coronary artery occlusion was widely recognized as the most important determinant for cardiomyocyte death from ischemic heart disease, substantial evidence indicated that reperfusion injury secondary to restored blood flow accounted for nearly half of infarct size, finally exacerbating ventricular dysfunction during ST-segment elevation myocardial infarction [1, 2]. In the past decades, the pathogenesis of myocardial I/R injury was deeply investigated, e.g., excessive generation of reactive oxygen species, calcium overload, mitochondrial permeability transition pore opening, mitochondrial dysfunction, platelet aggregation, and apoptosis were partially responsible for the cardiomyocyte loss under reperfusion damage [17, 18]. Additionally, autophagy which recycled the impaired organelles, e.g., mitochondria or misfolded protein, for cardiac cellular homeostasis and ATP supply was implicated in the pathological process of myocardial I/R injury [4, 19]. Notably, either excessive or insufficient autophagy was associated with increased cell death, thus resulting in an enlarged infarct area and compromised ventricular contraction. Moreover, both *in vivo* and *in vitro* evidence demonstrated that plasma exosomes (particularly rich in miRNA or proteins) exerted cardioprotection against severe injury during ischemia/reperfusion mediated by activation of the HSP70/TLR4 communication axis [20]. Prior studies had identified that miRNAs were important targets for regulating myocardial reperfusion damage [21, 22]. More recently, lncRNAs had also been extensively investigated in the context of ischemic heart disease, especially as key mediators of acute myocardial I/R injury and also targets for cardioprotection [23]. Emerging evidence showed a mutual interaction between lncRNAs and miRNAs; lncRNAs were demonstrated as a sponge to inhibit the expression of miRNA, regulating the activity of miRNA, along with competing for mRNAs. Among the aforementioned pathogenesis of myocardial tissue damage during reperfusion insult, oxidative stress was the most well-established basic mechanism and characterized by severe imbalance between exaggerated ROS generation and corresponding antioxidant defense systems [5]. In the context of myocardial I/R injury, upregulated expression of NOXs in infiltrated neutrophils, eNOS uncoupling, and impaired mitochondria were the main source of ROS which further resulted in mitochondrial damage, triggering the caspase-dependent apoptotic pathway. Moreover, ROS was considered to be a major determinant in adverse ventricular remodeling via promoting interstitial fibrosis, deposition of collagen, cardiomyocyte hypertrophy, or induction of cell death [24]. It thus provided the rationale for developing therapeutic options against the vicious cycle of ROS synthesis and degradation. Nonetheless, effective interventions which could translate into clinical application remained lacking due to the conflicting data between experimental evidence and human clinical trials.

Melatonin, a key factor in controlling the circadian rhythm, had been demonstrated to play a pivotal role in various cardiovascular diseases including heart failure, atherosclerosis, myocardial infarction, hypertension, vascular endothelial dysfunction, or cardiotoxicity [25]. Substantial evidence showed that the cardioprotective action of melatonin was closely related to its antioxidant properties [26]. Meanwhile, recent studies indicated a profound beneficial effect of melatonin against oxidative stress and retarding the deterioration of cardiac function after myocardial I/R injury [10, 11]. Early in 2004, Sahna and colleagues found that melatonin administration significantly inhibited the expression of MDA and increased the GSH levels in a mouse myocardial I/R model [27]. Subsequently, Yu et al. showed a significant antioxidative stress effect in retarding the surge of NADPH oxidase (gp91^phox^) and MDA, accompanied by a restored level of SOD after reperfusion damage [28]. Recently, experimental researches further confirmed the role of melatonin as a powerful free radical scavenger, which contributed to preserved contractile function and reduced infarct area in the context of myocardial I/R insult [10]. Unsurprisingly, this meta-analysis presented the outstanding therapeutic properties of melatonin in attenuating the infarct size post I/R injury, which therefore may account for the improved cardiac function (i.e., EF and FS). However, convincing experimental data outlined the other cell biological actions implicated in melatonin's beneficial effects, for instance, anti-inflammation, antiapoptosis, and regulating mitochondrial function and autophagy, as well as modulating the metabolic process [9, 29, 30]. Moreover, Genade et al. indicated that the antiadrenergic effects mediated by NO and RISK pathway were also responsible for melatonin-induced cardioprotection against I/R damage [12]. Noteworthily, further mechanistic explorations found that there were various crucial downstream signaling pathways underlying the favorable effects of melatonin in the treatment of cardiovascular disease. Previous studies reported that the activation of the Nrf2 pathway is a key antioxidant mediator in melatonin treatment by enhancing the expression of HO-1, GAPDH, or GSH S-transferase a-1 (GST-a1). In addition, the ROS-scavenging system was activated by melatonin through Notch1/Hes1/Akt pathways which maintained the intracellular redox homeostasis [31]. Furthermore, there was also a direct corelationship between the JAK/STAT signaling pathway and melatonin receptor, which facilitated the antioxidative stress processes in the reperfused heart [25]. Pretreatment with melatonin by Yang et al. showed increased expression of SOD and suppressed generation of MDA and mitochondrial H_2_O_2_ by activation of JAK2/STAT3 signaling cascade in perfused isolated hearts, whereas it was abolished in the presence of AG490 (JAK2/STAT3 inhibitor) or genetic modulation (JAK2 siRNA) [32]. Meanwhile, activation of JAK2/STAT3 provided additional antiapoptotic effects by regulating the expression of Bcl-2/Bax. Importantly, melatonin was also implicated in the autophagic process during the reperfusion damage via the AMPK/mTOR signaling pathway [33]. Recent studies highlighted the decisive role of PPAR*γ*/FUNDC1 and AMPK*α* axis which modulated the function and structure of mitochondria (i.e., mitophagy, mitochondrial fission, HK2-VDAC1 interaction, or mitochondria-induced apoptotic pathway) in the therapeutic action of melatonin [13]. Overall evidence from basic researches showed that melatonin treatment was a promising cardioprotective strategy in the context of myocardial I/R injury.

However, the published data on the efficacy of melatonin in STEMI patients receiving early revascularization remained lacking. A nested case-control study found that low melatonin secretion was expressively associated with a higher risk of myocardial infarction in women with increased BMI [34]. Unexpectedly, the MARIA trial demonstrated that melatonin administration did not reduce the infarct size; contrariwise, it may aggravate ventricular remodeling [35]. Nevertheless, subsequent post hoc analysis revealed that early treatment with melatonin resulted in less infarct area in patients suffering from reperfusion damage [36]. In addition, Dwaich et al. found a dose-dependent protective effect of melatonin in suppressing the pathological process during the CABG [37]. Thus, the different dosages and duration of ischemia among the clinical studies may be responsible for the conflicting results. Divergent plasma concentration of melatonin, mass of salvage myocardium after I/R insult, and even the distinct activated signaling cascades in different pathological phases collectively weakened the beneficial effect of melatonin [38, 39]. Meanwhile, the timing and form of drug delivery and approaches for infarct size determination may also have impact on the outcomes. Altogether, so far, it was quite challenging to translate the favorable effects of melatonin into the clinical setting. More well-designed RCTs were pressing need to further identify the cardioprotective role of melatonin in myocardial I/R injury.

### 4.1. Limitation

First, the results of our meta-analysis were based on study-level data rather than individual animal-level data which impeded further subgroup analysis, i.e., detailed dosage of melatonin treatment, precise age, or body weight of each rodent that may have an impact on pharmacokinetic or pharmacodynamic profile of melatonin intake, along with laboratory mouse or rat strains. Second, there was no standard regimen about the timing of either ischemic/reperfusion duration or melatonin precondition. For instance, the huge difference of time interval for melatonin pretreatment (ranged from 10 minutes to 4 weeks) may also confuse the most optimal administration time of melatonin, despite the remarkable consistency across all the included studies. Third, significant high heterogeneity in this work may inevitably affect the interpretation of results; however, robust data evidenced by both sensitivity analysis and stratified analysis verified the benefits and reliability of melatonin treatment in ameliorating the infarct size post reperfusion injury. In accordant with this, metaregression also failed to reveal any influence of prespecified covariates on pooled estimates of infarct size. Fourth, it was regrettable that overall included studies reported the instant efficacy of melatonin in improving infarct size and inhibiting subsequent cardiac dysfunction, but whether pretreatment with melatonin could maintain its cardioprotective effect for a long while was unknown and needed further exploration. Finally, there was a critical weakness in our work that the evidence of favorable effects with melatonin precondition was not confirmed by large animal studies (more relevant to humans). Therefore, there was pressing need for further investigations in large animals before the human clinical trials.

## 5. Conclusion

Melatonin treatment was associated with a significant improvement in infarct size and cardiac function in rodent hearts post I/R injury. It provided the rationale for clinical application of melatonin combined with immediate coronary revascularization in acute myocardial infarction patients.

## Figures and Tables

**Table 1:** Baseline characteristics of studies, animals, and melatonin treatment.

Author	Year	State	Species	Weight/year	Type of I/R	Anesthetic	Sample size		I/R duration	Infarct size/AAR	Melatonin treatment
							Control	Melatonin			
Chen et al.[31]	2018	China	Rats, SD	250-300 g	In vivo	Chloral hydrate	6	6	30 min/2 h	Evans blue/TTC	20 mg/kg, i.p., 12 h before I/R
Zhou et al.[13]	2017	China	Mice, C57BL/6	20-25 g	In vivo	Pentobarbital sodium	6	6	120 min/4 h	Evans blue/TTC	20 mg/kg, i.p., 12 h before I/R
Zhai et al. [10]	2017	China	Mice, C57BL/6	20-22 g	In vivo	2% isoflurane	8	8	30 min/24 h	Evans blue/TTC	20 mg/kg, i.p., 10 min before I/R
Zhou et al. [17]	2017	China	Mice, C57BL/6	20-25 g	In vivo	NA	6	6	30 min/2 h	Evans blue/TTC	20 mg/kg, i.p., 12 h before I/R
He et al. [9]	2016	China	Mice, C57BL/6	NA	In vivo	2% isoflurane	8	8	30 min/24 h	Evans blue/TTC	150 *μ*g/kg, i.p., 30 min before I/R
Yu et al. [29]	2015	China	Rats, SD	200-250 g	In vivo	3% pentobarbital sodium	8	8	30 min/6 h	Evans blue/TTC	10 mg/kg, p.o., 4 weeks before I/R
Yu et al. [26]	2014	China	Rats, SD	200-220 g	In vivo	3% pentobarbital sodium	6	6	30 min/6 h	Evans blue/TTC	10 mg/kg, i.p., 7 days before I/R
Chen et al. [27]	2009	China	Mice, C57BL/6	4-5 months	In vivo	Tribromoethanol	6	6	50 min/4 h	Evans blue/TTC	150 *μ*g/kg, i.p., 30 min before I/R
Genade et al. [12]	2008	South Africa	Rats, Wistar	230–280 g	Ex vivo	Pentobarbital sodium	7	11	35 min/2 h	Evans blue/TTC	50 *μ*M, perfusion, 10 min before I/R
Lochner et al. [24]	2006	South Africa	Rats, Wistar	220–250 g	Ex vivo	Pentobarbital sodium	6	6	35 min/2 h	Evans blue/TTC	50 *μ*M, perfusion, 10 min before I/R
Sahna et al. [25]	2005	Turkey	Rats, Wistar	250-300 g	In vivo	Urethane	8	8	30 min/2 h	Fluorescent particles/TTC	10 mg/kg, i.v., 10 min before I/R
Sahna et al. [11]	2002	Turkey	Rats, Wistar	150-200 g	In vivo	Urethane	8	8	30 min/2 h	Fluorescent particles/TTC	4 mg/kg, i.v., 10 min before I/R
Lee et al. [14]	2002	China	Rats, SD	250–300 g	In vivo	Pentobarbital sodium	6	6	45 min/1 h	Methylene blue/TTC	5 mg/kg, i.v., 10 min before I/R
Lagneux et al. [35]	2000	France	Rats, Wistar	280–350 g	Ex vivo	Sodium pentobarbital	6	6	30 min/2 h	Blue dye/TTC	10 mg/kg, i.p., 30 min before I/R
Nduhirabandi et al. [28]	2010	South Africa	Rats, Wistar	180-220 g	Ex vivo	Sodium pentobarbital	8	9	40 min/2 h	Evans blue/TTC	4 mg/kg, p.o., 16 weeks before I/R

SD: Sprague-Dawley rats; M: male; i.p.: intraperitoneal injection; i.v.: intravenous injection; p.o.: orally treated; I/R: ischemia/reperfusion injury; TTC: triphenyltetrazolium chloride.

**Table 2:** The quality of included studies.

Studies	Year	A	B	C	D	E	F	Score
Chen et al.	2018	Y	Y	N	N	Y	Y	4
Zhou et al.	2017	Y	N	N	N	Y	Y	3
Zhai et al.	2017	Y	Y	N	N	Y	Y	4
Zhou et al.	2017	Y	N	N	N	Y	Y	3
He et al.	2016	Y	N	N	N	Y	Y	3
Yu et al.	2015	Y	Y	N	N	Y	Y	3
Yu et al.	2014	Y	Y	N	N	Y	Y	4
Chen et al.	2009	Y	N	N	N	Y	Y	3
Genade et al.	2008	Y	N	N	N	Y	N	2
Lochner et al.	2006	Y	N	N	N	Y	N	2
Sahna et al.	2005	Y	N	N	N	Y	N	2
Sahna et al.	2002	Y	Y	N	N	Y	Y	4
Lee et al.	2002	Y	Y	N	N	Y	Y	4
Lagneux et al.	2000	Y	N	N	N	Y	N	2
Nduhirabandi et al.	2010	Y	N	N	N	Y	Y	3

A: peer-reviewed publication; B: random allocation to groups; C: blinded assessment of outcomes; D: sample size calculation; E: compliance with animal welfare regulations; F: a statement of a potential conflict of interest; Y: yes; N: no.

**Table 3:** The molecular and cellular mechanisms underlying the cardioprotection of melatonin treatment in myocardial I/R injury.

Studies	Year	Proposed mechanisms
Chen et al.	2018	Inhibit autophagy via AMPK/mTOR signaling pathway
Zhou et al.	2017	Attenuate FUNDC1-required mitophagy, inflammation, improve microvascular function via regulating the expression of platelet PPAR*γ*
Zhai et al.	2017	Antioxidative stress, antiapoptosis through activation of SIRT3 signaling pathway
Zhou et al.	2017	Regulate mitochondrial fission, mitophagy, mPTP opening, and HK2-VDAC1 interaction
He et al.	2016	Restore autophagy function, suppress oxidative stress and apoptosis through nuclear receptor ROR*α*
Yu et al.	2015	Notch1/Hes1 signaling and Pten/Akt signaling underlie the antioxidative stress and antiapoptosis effect
Yu et al.	2014	Reduce apoptosis and oxidative damage via SIRT1 signaling
Chen et al.	2009	Attenuate apoptosis independent of Gpx1
Genade et al.	2008	Antiadrenergic actions mediated by NO and PKC signaling, PKB/Akt activation, and p38MAPK signaling involved in the cardioprotection
Lochner et al.	2006	NA
Sahna et al.	2004	Attenuate oxidative stress (reduce MDA and restore GSH level)
Sahna et al.	2002	NA
Lee et al.	2002	Antioxidant activity, inhibit neutrophil infiltration
Lagneux et al.	2000	NA
Nduhirabandi et al.	2010	Prevent metabolic abnormality via modulating insulin release and PKB/Akt and ERK 1/2 signaling

**Table 4:** Sensitivity analysis.

Study omitted	Year	WMD	95% CI	*p* value	Heterogeneity
Chen et al.	2018	-20.00	-25.29, -14.70	<0.001	*I* ^2^ = 91.6%, *p* < 0.001
Zhou et al.	2017	-19.76	-24.80, -14.71	<0.001	*I* ^2^ = 91.8%, *p* < 0.001
Zhai et al.	2017	-20.83	-26.14, -15.51	<0.001	*I* ^2^ = 91.9%, *p* < 0.001
Zhou et al.	2017	-18.80	-23.01, -14.59	<0.001	*I* ^2^ = 86.2%, *p* < 0.001
He et al.	2016	-21.30	-26.40, -16.21	<0.001	*I* ^2^ = 90.5%, *p* < 0.001
Yu et al.	2015	-20.80	-26.00, -15.59	<0.001	*I* ^2^ = 91.9%, *p* < 0.001
Yu et al.	2014	-20.74	-25.92, -15.52	<0.001	*I* ^2^ = 92.0%, *p* < 0.001
Chen et al.	2009	-20.32	-25.45, -15.19	<0.001	*I* ^2^ = 92.0%, *p* < 0.001
Genade et al.	2008	-20.75	-26.40, -15.10	<0.001	*I* ^2^ = 91.8%, *p* < 0.001
Lochner et al.	2006	-19.61	-24.65, -14.58	<0.001	*I* ^2^ = 90.3%, *p* < 0.001
Sahna et al.	2005	-20.85	-26.08, -15.62	<0.001	*I* ^2^ = 91.9%, *p* < 0.001
Sahna et al.	2002	-21.21	-26.32, -16.10	<0.001	*I* ^2^ = 91.8%, *p* < 0.001
Lee et al.	2002	-19.70	-24.48, -14.56	<0.001	*I* ^2^ = 91.6%, *p* < 0.001
Lagneux et al.	2000	-21.32	-26.42, -16.23	<0.001	*I* ^2^ = 90.6%, *p* < 0.001
Nduhirabandi et al.	2010	-20.72	-26.29, -15.14	<0.001	*I* ^2^ = 91.9%, *p* < 0.001
Overall		-20.45	-25.43, -15.47	<0.001	<0.001

**Table 5:** *Post hoc* subgroup analysis of pooled estimates of infarct size.

Pooled estimates	No. of studies	WMD	95% CI	*p* value	Heterogeneity
Species					
Rats	10	-19.06	-23.87, -14.25	<0.001	*I* ^2^ = 87.3%, *p* < 0.001
Mice	5	-24.83	-39.20, -10.46	<0.001	*I* ^2^ = 95.6%, *p* < 0.001
Study type					
*In vivo*	11	-21.27	-28.50, -14.04	<0.001	*I* ^2^ = 90.3%, *p* < 0.001
*Ex vivo*	4	-18.76	-26.22, -11.29	<0.001	*I* ^2^ = 93.8%, *p* < 0.001
Reperfusion duration					
≥6 h	11	-22.84	-28.89, -16.78	<0.001	*I* ^2^ = 92.3%, *p* < 0.001
<6 h	4	-13.04	-16.40, -9.68	<0.001	*I* ^2^ = 15.0%, *p* = 0.317
Timing regimen of pretreatment					
>60 min	6	-24.89	-33.87, -15.92	<0.001	*I* ^2^ = 91.6%, *p* < 0.001
≤60 min	9	-17.68	-23.45, -11.92	<0.001	*I* ^2^ = 89.8%, *p* < 0.001
Overall	15	-20.45	-25.43, -15.47	<0.001	*I* ^2^ = 91.4%, *p* < 0.001

**Table 6:** Metaregression of pooled estimates of infarct size.

Covariates	Infarct size		
	Coefficient	95% CI	*p* value
Sample size	2.360435	0.3001453, 4.420724	0.208
Species	-5.111393	-17.30782, 7.085032	0.382
Study type	2.456891	-9.851739, 14.76552	0.673
State	4.689403	-0.6606213, 10.03943	0.081
Route of administration	0.5208883	-4.255456, 5.297233	0.817
Duration of reperfusion	0.2287274	-3.982206, 4.439661	0.908
Timing regimen of pretreatment	3.072655	-0.4711795, 6.61649	0.084

## References

[1] Hausenloy D. J., Yellon D. M. (2013). Myocardial ischemia-reperfusion injury: a neglected therapeutic target. *The Journal of Clinical Investigation*.

[2] Turer A. T., Hill J. A. (2010). Pathogenesis of myocardial ischemia-reperfusion injury and rationale for therapy. *The American Journal of Cardiology*.

[3] Davidson S. M., Ferdinandy P., Andreadou I. (2019). Multitarget strategies to reduce myocardial ischemia/reperfusion injury: JACC review topic of the week. *Journal of the American College of Cardiology*.

[4] Ma S., Wang Y., Chen Y., Cao F. (2015). The role of the autophagy in myocardial ischemia/reperfusion injury. *Biochimica et Biophysica Acta*.

[5] Cadenas S. (2018). ROS and redox signaling in myocardial ischemia-reperfusion injury and cardioprotection. *Free Radical Biology & Medicine*.

[6] Cipolla-Neto J., do Amaral F. G. (2018). Melatonin as a hormone: new physiological and clinical insights. *Endocrine Reviews*.

[7] Dominguez-Rodriguez A., Abreu-Gonzalez P., Reiter R. J. (2009). Clinical aspects of melatonin in the acute coronary syndrome. *Current Vascular Pharmacology*.

[8] Dominguez-Rodriguez A., Abreu-Gonzalez P., Sanchez-Sanchez J. J., Kaski J. C., Reiter R. J. (2010). Melatonin and circadian biology in human cardiovascular disease. *Journal of Pineal Research*.

[9] He B., Zhao Y., Xu L. (2016). The nuclear melatonin receptor ROR*α*is a novel endogenous defender against myocardial ischemia/reperfusion injury. *Journal of Pineal Research*.

[10] Zhai M., Li B., Duan W. (2017). Melatonin ameliorates myocardial ischemia reperfusion injury through SIRT3-dependent regulation of oxidative stress and apoptosis. *Journal of Pineal Research*.

[11] Sahna E., Acet A., Ozer M. K., Olmez E. (2002). Myocardial ischemia-reperfusion in rats: reduction of infarct size by either supplemental physiological or pharmacological doses of melatonin. *Journal of Pineal Research*.

[12] Genade S., Genis A., Ytrehus K., Huisamen B., Lochner A. (2008). Melatonin receptor-mediated protection against myocardial ischaemia/reperfusion injury: role of its anti-adrenergic actions. *Journal of Pineal Research*.

[13] Zhou H., Li D., Zhu P. (2017). Melatonin suppresses platelet activation and function against cardiac ischemia/reperfusion injury via PPAR*γ*/FUNDC1/mitophagy pathways. *Journal of Pineal Research*.

[14] Lee Y. M., Chen H. R., Hsiao G., Sheu J. R., Wang J. J., Yen M. H. (2002). Protective effects of melatonin on myocardial ischemia/reperfusion injury in vivo. *Journal of Pineal Research*.

[15] Macleod M. R., O’Collins T., Howells D. W., Donnan G. A. (2004). Pooling of animal experimental data reveals influence of study design and publication bias. *Stroke*.

[16] Macleod M. R., O'Collins T., Horky L. L., Howells D. W., Donnan G. A. (2005). Systematic review and meta-analysis of the efficacy of melatonin in experimental stroke. *Journal of Pineal Research.*.

[17] Heusch G., Gersh B. J. (2017). The pathophysiology of acute myocardial infarction and strategies of protection beyond reperfusion: a continual challenge. *European Heart Journal*.

[18] Petrosillo G., Di Venosa N., Moro N. (2011). In vivo hyperoxic preconditioning protects against rat-heart ischemia/reperfusion injury by inhibiting mitochondrial permeability transition pore opening and cytochrome c release. *Free Radical Biology & Medicine*.

[19] Zhou H., Zhang Y., Hu S. (2017). Melatonin protects cardiac microvasculature against ischemia/reperfusion injury via suppression of mitochondrial fission-VDAC1-HK2-mPTP-mitophagy axis. *Journal of Pineal Research*.

[20] Vicencio J. M., Yellon D. M., Sivaraman V. (2015). Plasma exosomes protect the myocardium from ischemia-reperfusion injury. *Journal of the American College of Cardiology*.

[21] Ren X.-P., Wu J., Wang X. (2009). MicroRNA-320 is involved in the regulation of cardiac ischemia/reperfusion injury by targeting heat-shock protein 20. *Circulation*.

[22] Aurora A. B., Mahmoud A. I., Luo X. (2012). MicroRNA-214 protects the mouse heart from ischemic injury by controlling Ca2+ overload and cell death. *The Journal of Clinical Investigation*.

[23] Ong S. B., Katwadi K., Kwek X. Y. (2018). Non-coding RNAs as therapeutic targets for preventing myocardial ischemia-reperfusion injury. *Expert Opinion on Therapeutic Targets*.

[24] Munzel T., Camici G. G., Maack C., Bonetti N. R., Fuster V., Kovacic J. C. (2017). Impact of oxidative stress on the heart and vasculature: part 2 of a 3-part series. *Journal of the American College of Cardiology*.

[25] Yang Y., Sun Y., Yi W. (2014). A review of melatonin as a suitable antioxidant against myocardial ischemia-reperfusion injury and clinical heart diseases. *Journal of Pineal Research*.

[26] Lochner A., Marais E., Huisamen B. (2018). Melatonin and cardioprotection against ischaemia/reperfusion injury: what's new? A review. *Journal of Pineal Research*.

[27] Sahna E., Parlakpinar H., Turkoz Y., Acet A. (2005). Protective effects of melatonin on myocardial ischemia/reperfusion induced infarct size and oxidative changes. *Physiological Research*.

[28] Yu L., Sun Y., Cheng L. (2014). Melatonin receptor-mediated protection against myocardial ischemia/reperfusion injury: role of SIRT1. *Journal of Pineal Research*.

[29] Chen Z., Chua C. C., Gao J. (2009). Prevention of ischemia/reperfusion-induced cardiac apoptosis and injury by melatonin is independent of glutathione peroxdiase 1. *Journal of Pineal Research*.

[30] Nduhirabandi F., Du Toit E. F., Blackhurst D., Marais D., Lochner A. (2011). Chronic melatonin consumption prevents obesity-related metabolic abnormalities and protects the heart against myocardial ischemia and reperfusion injury in a prediabetic model of diet-induced obesity. *Journal of Pineal Research*.

[31] Yu L., Liang H., Lu Z. (2015). Membrane receptor-dependent Notch1/Hes1 activation by melatonin protects against myocardial ischemia-reperfusion injury: in vivo and in vitro studies. *Journal of Pineal Research*.

[32] Yang Y., Duan W., Jin Z. (2013). JAK2/STAT3 activation by melatonin attenuates the mitochondrial oxidative damage induced by myocardial ischemia/reperfusion injury. *Journal of Pineal Research*.

[33] Zhu H., Jin Q., Li Y. (2018). Melatonin protected cardiac microvascular endothelial cells against oxidative stress injury via suppression of IP3R-[Ca2+]c/VDAC-[Ca2+]m axis by activation of MAPK/ERK signaling pathway. *Cell Stress and Chaperones*.

[34] McMullan C. J., Rimm E. B., Schernhammer E. S., Forman J. P. (2017). A nested case-control study of the association between melatonin secretion and incident myocardial infarction. *Heart*.

[35] Dominguez-Rodriguez A., Abreu-Gonzalez P., de la Torre-Hernandez J. M. (2017). Effect of intravenous and intracoronary melatonin as an adjunct to primary percutaneous coronary intervention for acute ST-elevation myocardial infarction: results of the melatonin adjunct in the acute myocardial infarction treated with angioplasty trial. *Journal of Pineal Research*.

[36] Dominguez-Rodriguez A., Abreu-Gonzalez P., de la Torre-Hernandez J. M. (2017). Usefulness of early treatment with melatonin to reduce infarct size in patients with ST-segment elevation myocardial infarction receiving percutaneous coronary intervention (from the melatonin adjunct in the acute myocardial infarction treated with angioplasty trial). *Amerian Journal Cardiology*.

[37] Dwaich K. H., Al-Amran F. G. Y., AL-Sheibani B. I. M., Al-Aubaidy H. A. (2016). Melatonin effects on myocardial ischemia–reperfusion injury: Impact on the outcome in patients undergoing coronary artery bypass grafting surgery. *International Journal of Cardiology*.

[38] Manintveld O. C., te Lintel Hekkert M., van den Bos E. J. (2007). Cardiac effects of postconditioning depend critically on the duration of index ischemia. *American Journal of Physiology Heart and Circulatory Physiology*.

[39] Ekeloef S., Halladin N., Fonnes S. (2017). Effect of intracoronary and intravenous melatonin on myocardial salvage index in patients with ST-elevation myocardial infarction: a randomized placebo controlled trial. *Journal of Cardiovascular Translational Research.*.

